# Impact of pediatric anesthesia management on cancer outcomes in children—a narrative review

**DOI:** 10.3389/fonc.2025.1621620

**Published:** 2025-07-04

**Authors:** Aileen Fenelon, Neil Hauser, Britta S. von Ungern-Sternberg

**Affiliations:** ^1^ Department of Anaesthesia and Pain Medicine, Perth Children’s Hospital, Perth, WA, Australia; ^2^ Institute for Paediatric Perioperative Excellence, The University of Western Australia, Perth, WA, Australia; ^3^ Division of Emergency Medicine, Anaesthesia and Pain Medicine, Medical School, The University of Western Australia, Perth, WA, Australia; ^4^ Perioperative Medicine Team, Perioperative Care Program, The Kids Research Institute Australia, Perth, WA, Australia

**Keywords:** pediatric, child, cancer, outcomes, anaesthesia, anesthesia - general

## Abstract

**Importance:**

The relationship between anesthetic technique and pediatric oncological outcomes is an emerging field of interest. With significant improvements in childhood cancer survival in recent decades, there is an increased focus on optimizing the quality of survival and reducing the incidence of metastasis and recurrence. The aim of this narrative review article is to investigate and consolidate the current available evidence assessing the immunomodulatory effects of anesthesia in the pediatric oncology population.

**Observations:**

There is mounting evidence supporting an association between perioperative interventions such as anesthetic techniques and oncological outcomes in adults. Research, predominantly based on laboratory studies and retrospective studies in the adult population, has explored this association, often with mixed results. Some studies found that agents such as volatile anesthetics promoted cancer cell dissemination or recurrence by altering tumor microenvironments, while others argued that the influence of anesthesia on cancer recurrence is minimal and emphasized the need for further, more targeted research.

**Conclusions and relevance:**

The significant differences which exist between adult and pediatric oncology populations, in terms of immune system maturation, underlying malignancy, treatment regimens, and frequency of anesthetic exposure, present a further challenge in applying the findings from the current, mainly adult-based evidence to pediatric anesthesia practice. Evidence suggests a trend toward an effect rather than a definitive answer. A large, high-quality randomized, controlled trial is warranted to further our understanding of the effects of anesthesia in pediatric oncology patients.

## Introduction

The Australian Childhood Cancer Registry reports that, on average, approximately 810 children, aged 0–14 years old, are diagnosed with cancer in Australia each year, and 45% of them were of ages 0–4 years old (1). The National Cancer Institute reports similar statistics for childhood cancer in the United States, estimating that 9,620 children aged 0–14 years will be diagnosed with cancer in 2024 (2).

Cancer survival in children has improved significantly with overall 5-year survival rates of 86% in Australia (1). As a result of high survival rates, the quality of survival has become increasingly important. Cancer treatment modalities including surgery, chemotherapy, radiotherapy, and immunotherapy may result in long-term, multi-organ chronic health conditions (1). Cancer treatment in children can also result in significant psychological and financial burdens on patients and their families. Prevention of both metastases and recurrence in pediatric cancers is an evolving and essential strategy in reducing mortality and optimizing outcomes.

The impact of perioperative interventions, particularly the type of anesthesia administered, on cancer recurrence and survival in adults has been widely considered (3, 4). Given the increased frequency of exposure to anesthesia in children, in addition to the potential longer period for cancer recurrence, and the personal and financial implications of cancer-related morbidity, any interaction between anesthesia and pediatric oncological outcomes is vital to consider.

The three most common types of childhood cancer are acute lymphoid leukemia (24%), astrocytoma (10%), and neuroblastoma (6%) (1). This contrasts with cancers in adults in which solid organ tumors predominate (5). As a result, there is significant heterogeneity in the treatment modalities and outcomes between adults and children. Data obtained through studies in the adult population may have little, if any, application to children.

Consideration must also be given for confounding factors when studying the impacts of anesthesia on both tumor growth and recurrence. There is considerable variability in tumor size and growth, toxic effects of chemotherapy and radiation therapy, and their interaction with anesthetic agents as well as the heterogeneity of the pediatric population which includes neonates through to adolescents, and the presence of complications from cancers including sepsis, thromboembolism, tumor lysis syndrome, and multi-organ failure.

Pediatric anesthetists are regularly involved in the care of pediatric oncology patients, including the facilitation of treatment, vascular access, imaging, and surgical resection. Therefore, it is necessary to be aware not only of the continually evolving changes in treatment but also any impact that anesthetic technique may have on patients’ long-term outcomes, particularly given that children commonly have a high cumulative burden of anesthetic exposure over a relatively short period of time.

This review article investigates the currently available evidence assessing the immunomodulatory effects of anesthesia in the pediatric oncology population. Where that information is unavailable, we discuss the applicability or correlation of data extrapolated from the adult population.

## Pathophysiology

Cancer is described as the unregulated proliferation of abnormal cells. Tumor initiation is caused by a genetic alteration resulting in an abnormal cell. Unregulated proliferation then occurs due to abnormalities of multiple cell regulatory systems. Cancers can be classified as carcinomas—malignancies of epithelial cells, sarcomas—solid tumors of connective tissues, and leukemias/lymphomas—malignancies of hemopoietic cells or cells of the immune system.

The innate and adaptive immune systems act together to protect the body against tumor growth. The components of the innate immune system that directly target cancer cells include natural killer cells, dendritic cells, macrophages, polymorphonuclear leukocytes, mast cells, and cytotoxic T-lymphocytes. The innate immune system activates the adaptive immune system through the activation of T-cells and B-cells (6). Abnormalities in immune regulatory mechanisms prevent the natural immune response of selectively killing cancer cells. The developing tumor induces an inflammatory state, which is conducive to the recruitment of immune cells. These immune cells typically do not exhibit the normal protective anti-tumor response and may release pro-inflammatory cytokines, resulting in tumor progression (7).

Anesthetic agents are often administered during a vulnerable period in the establishment of metastasis. During surgical resection of a solid tumor, neoplastic cells may inadvertently be dispersed into the lymphatic system and circulation due to tissue damage and tumor handling, thereby establishing metastases. The neuroendocrine and cytokine stress response associated with a major surgery and the effects of cancer treatment modalities may result in the transient suppression of cell-mediated immunity, creating an immune system that is susceptible to further influences from anesthetic agents and/or techniques (7). Cancer recurrence or metastases depend on both the presence of residual cancer cells and the ability of those residual cancer cells to escape the host’s immune system response (4). Several postoperative changes have been described, which may aid the survival of cancer cells in the circulation. Experimental models have shown that reduced natural killer cytotoxicity and impaired macrophage function were both associated with increased tumor growth after surgery (8).

Hematological malignancies predominate in children. While they may not require an extensive surgical resection, with a prolonged general anesthetic and exposure to the associated neuroendocrine stress response, children often require several interventions necessitating repeated general anesthetics. This cumulative burden, even in the absence of surgical resection, can be significant with numerous radiotherapy or chemotherapy treatments, imaging, and vascular access procedures. This is an important consideration for our evaluation of the current evidence and the impact of anesthesia on oncological outcomes.

## Inhalational versus total intravenous anesthesia

Volatile anesthetics affect many organ systems, including the immune system (3). Volatiles suppress the innate immune system by reducing circulating neutrophils, dendritic cells, natural killer cells, and tissue macrophages (9). They also cause suppression of the adaptive immune system by limiting the proliferation of lymphocytes (9). In addition, volatiles have been shown to indirectly modulate the immune system through their enhanced effect on stress hormone release (10). In cancer patients, it has been proposed that this volatile-mediated immunosuppression contributes to tumor growth, recurrence, and metastases (9). Several studies have shown that volatile anesthetics increase the expression of prometastatic and protumorigenic factors on tumor cells via signaling pathways, including hypoxia inducible factor-1-alpha and transforming growth-factor-beta (11–14).

In contrast, propofol mainly has an anti-tumor effect, predominantly occurring through reduced cancer cell proliferation and invasion and increased cancer cell apoptosis through the downregulation of matrix metalloproteinases and various immunosuppressing cytokines (14–16).

Most of the current research assessing the impact of anesthesia on tumor growth and recurrence are based on retrospective studies in adults, animal models, or *in vitro* studies. This data shows a trend toward a beneficial effect of propofol-based TIVA outcomes, specifically overall survival and recurrence rates (3). There are, however, important considerations assessing the applicability of the currently available evidence to children. Pediatric oncology patients often undergo many general anesthetics to facilitate treatment, vascular access, or imaging over a relatively short period of time. While the duration of anesthesia is often short, the cumulative effect may be significant. Additionally, particularly in the younger pediatric oncology patient cohort, repeated interventions may result in significant periprocedural anxiety and distress, which may perpetuate the sympathetic stress response and further influence immune surveillance (17). Furthermore, studies often consider anesthetic agents as if used alone, while in clinical practice they are almost always used in combination with other medications such as opioids.

A systematic review and meta-analysis of 19 retrospective observational studies in adults determined that patients receiving propofol-based TIVA during cancer surgery had significantly better overall survival than those receiving volatile anesthesia (HR: 0.79, 95% CI: 0.66–0.94, *p* = 0.008). There was, however, no statistically significant difference in recurrence-free survival between groups (HR: 0.81, 95% CI: 0.61–1.07, *p* = 0.137). In the subgroup analysis, patients in the TIVA group had better overall survival compared to those receiving desflurane, but there was no statistically significant difference in overall survival when compared to those receiving sevoflurane (14).

A retrospective analysis of over 7,000 adults undergoing cancer surgery found that patients had a worse outcome if they received volatile anesthesia irrespective of their ASA score, surgical severity, or whether they had recorded metastases at the time of surgery. In 2,607 propensity-score-matched patients, overall mortality was 22.8% with volatile and 11.2% with TIVA, with an adjusted hazard ratio of 1.46 (95% CI: 1.29 to 1.66) (18).

A further retrospective analysis of patients undergoing a modified radical mastectomy for breast cancer found no difference in overall survival between patients receiving TIVA or sevoflurane-based anesthesia but a lower rate of cancer recurrence in the propofol group (19). A multi-center randomized controlled trial of 1,764 breast cancer patients similarly found no difference in overall survival between propofol or sevoflurane groups (20).

A meta-analysis of TIVA versus volatile anesthesia in adults with breast, esophageal, and non-small cell lung cancer found that the use of TIVA was associated with improved recurrence-free survival in all cancer types (pooled HR: 0.78, 95% CI: 0.65–0.94, *p* < 0.01) and improved overall survival (pooled HR: 0.76, 95% CI: 0.63–0.92, *p* < 0.01) (21). These findings have been further supported by another retrospective observational study in adults with esophageal cancer (22).

A prospective randomized trial comparing the immunosuppressive effects of propofol compared to sevoflurane using blood samples collected from women undergoing breast cancer surgery determined that changes in immune cells were similar in both the propofol and sevoflurane groups (23). These findings are supported by a retrospective cohort study of over 7,000 patients who had breast cancer surgery which found that inhalational anesthesia had no significant impact on recurrence-free survival (HR: 0.96, 95% CI: 0.69–1.32, *p* = 0.782) or overall survival (HR: 0.96, 95% CI: 0.69–1.33, *p* = 0.805) when compared with TIVA (24). Additionally, these findings align with a national-registry-based study of 11,598 patients with stages 1–3 colorectal cancer (25). Similarly, a more recent study of 536 adults undergoing hepatectomy for hepatocellular carcinoma found that intraoperative anesthesia technique did not affect postoperative recurrence or overall survival. It is worth noting that in the subgroup undergoing open hepatectomy, there was a significantly lower risk of tumor recurrence or death identified in the TIVA group (HR: 0.49, 95% CI: 0.25–0.95, *p* = 0.034). This contrasts with the subgroup undergoing laparoscopic surgery, where no significant difference was observed (HR: 1.14, 95% CI: 0.73–1.80, *p* = 0.558) (26).

A study comparing the effects of various anesthetic agents (ketamine, thiopental, halothane, and propofol) on natural killer cell activity and on resistance to experimental metastasis found that all anesthetic agents, except propofol, significantly reduced natural killer activity and increased MADB106 lung tumor retention or lung metastases (27). **Table 1** summarizes the adult studies comparing volatile agents and TIVA.

**Table 1:** Summary of the adult studies comparing inhalation anesthesia agents and TIVA.

Study	Design	Intervention	Sample size	End points	Results
Chang et al. (14)	Systematic review and meta-analysis	Comparison of the effects of propofol-based TIVA and volatile anesthesia on long-term oncological outcomes in patients undergoing cancer surgery	19 retrospective observational studies 17 studies (23,489 patients) in a meta-analysis of overall survival (OS); 10 studies (8,980 patients) in a meta-analysis of recurrence-free survival (RFS)	Overall survival Recurrence-free survival Subgroup analysis comparing different volatile agents and different cancer types with OS and RFS	Propofol-based TIVA is associated with better OS than volatile anesthesia during cancer surgery. No statistically significant difference observed in RFS
Wigmore et al. (18)	Retrospective cohort study	Investigation of the association of anesthetic techniques with long-term survival in patients undergoing elective cancer surgery	3,316 patients in the inhalational group 3,714 patients in the TIVA group	1– year survival Long-term survival	1-year survival: TIVA group—94.1%, inhalational group—87.9% Overall mortality rate was 18.5% at 2.66 years in the TIVA group and 24% at 2.91 years in the inhalational group
Lee et al. (19)	Retrospective analysis of electronic medical records	Comparison of OS and RFS after modified radical mastectomy in patients with breast cancer who underwent propofol-based TIVA or sevoflurane-based anesthesia	Data from 325 patients analyzed—173 in the propofol group and 152 in the sevoflurane group	Recurrence-free survival Overall survival during the initial 5 years after surgery	Propofol group had a longer RFS (*p* = 0.037) than the sevoflurane group, but OS was not significantly different (*p* = 0.383)
Oh et al. (23)	Retrospective cohort study	Comparison of RFS and OS between propofol-based TIVA and inhalational agents when treating non-small cell lung cancer with curative resection	943 patients—749 in the TIVA group and 194 in the inhalational group	Postoperative recurrence-free survival and overall survival in both groups	No significant difference in the HR for recurrence between the TIVA and inhalational groups (*p* = 0.233) or death (*p* = 0.551)
Yap et al. (21)	Meta-analysis	Evaluation of the effects of TIVA and inhalational anesthetic agents on cancer outcomes in patients undergoing cancer surgery	1 RCT, 9 retrospective studies 6 studies (7,866 patients) in a meta-analysis of RFS 8 studies (18,778 patients) in a meta-analysis of OS	Overall survival Recurrence-free survival	Propofol TIVA may be associated with improved RFS and OS in patients having cancer surgery
Jun et al. (22)	Retrospective observational study	Evaluation of the association of general anesthesia agents with OS and RFS in patients who underwent esophageal cancer surgery	922 patients – 731 in the TIVA group, 191 in the volatile group	Overall survival and recurrence free survival in both groups	Volatiles were associated with worse overall survival and worse recurrence free survival
Yoo et al. (24)	Retrospective cohort study	Comparison of the influence of TIVA and inhalational anesthesia on recurrence-free survival after breast cancer surgery	5,331 patients—3,085 in the TIVA group and 2,246 in the inhalational group	Recurrence-free survival and overall survival after breast cancer surgery	No significant difference in RFS or OS 5-year RFS; rates of 93.2% in the TIVA group and 93.8% in the inhalational group

TIVA, total intravenous anesthesia; OS, overall survival; RFS, recurrence-free survival; RCT, randomized controlled trial.

Comparable data from pediatric oncology populations is scarce and severely lacking. One small study in the pediatric population compared the analgesic and anti-stress effects of inhalational and intravenous anesthesia in 49 children with various types of cancer undergoing tumor resection surgery. They studied the patients’ hemodynamic responses, level of cortisol, proinflammatory cytokines, and heart rate variability indicators and found no statistically significant difference in the level of intraoperative stress between the sevoflurane and propofol groups (28). It is worth noting that this is a small study with a very heterogenic cohort. Thus, it is unlikely to show statistically significant differences. In addition, this study did not report cancer outcomes.

A retrospective study performed on 150 pediatric patients with acute lymphoblastic leukemia (ALL) undergoing anesthesia for intrathecal chemotherapy found that the group receiving a combination of propofol and sevoflurane demonstrated greater recovery of T/B cell subset activity, thereby alleviating immunosuppression and impairing ALL progression, compared to the use of sevoflurane or propofol alone (29).

Furthermore, a small cohort study of 20 children with ALL undergoing bone marrow aspiration and lumbar puncture with methotrexate assessed the immunomodulatory activity of a combination of propofol and ketamine sedation on proinflammatory and anti-inflammatory cytokines. They found that propofol ketamine sedation had no effect on the plasma concentration of most measured cytokine levels and the T helper 1/2 ratio in children with ALL (30).

## Regional anesthesia

Regional anesthesia reduces the sympathetically driven surgical stress response and associated perioperative pain (3). Therefore, it has been hypothesized that regional anesthesia should minimize immunosuppression and has an anesthetic and analgesic-sparing effect with a beneficial effect on cancer outcomes. Additionally, *in vitro* studies have shown that amide local anesthetics, particularly the anti-inflammatory properties of lignocaine, may inhibit cancer cell activity and have beneficial effects on the immune system (31, 32).

A multi-center randomized controlled trial of 2,132 patients having potentially curative primary breast cancer resections found no difference in breast cancer recurrence between the paravertebral block and propofol group and the sevoflurane and opioids group (33). Similarly, a meta-analysis that included six RCTs investigating the effect of perioperative regional anesthesia in adults demonstrated no significant difference in the rate of cancer recurrence in patients receiving the adjunctive use of regional anesthesia compared to those receiving general anesthesia alone. However, the authors cautioned that these results were based on a low level of evidence (34).

Another systematic review and meta-analysis that included 15 RCTs, again based on an adult population, found that regional anesthesia did not have a positive effect on recurrence-free survival or overall survival when compared with general anesthesia (35).

In contrast, a retrospective review of medical records of patients with invasive prostate cancer who underwent an open radical prostatectomy found that the group who received an epidural in addition to general anesthesia had an estimated 57% (95% CI: 17%–78%) lower risk of recurrence compared with the group who received a general anesthetic and opioids with no epidural (HR: 0.43, 95% CI: 0.22–0.83, *p* = 0.012) (36).

Similarly to general anesthetic agents, there is a marked lack of evidence investigating the effects of regional anesthesia and cancer in the pediatric cohort.

A retrospective cohort study of 126 children undergoing primary resection of either neuroblastoma (51.6%), hepatoblastoma (13.5%), or sarcoma (34.9%) over 16 years investigated if an intraoperative epidural was associated with relapse-free survival in children with solid organ tumors (37). There was no statistically significant association between epidural use and improved relapse-free survival. In a subgroup (sarcoma group), there was a clinically meaningful lower risk of relapse with combined general anesthetic/epidural (37).

There remains, however, inadequate scientific evidence to demonstrate a beneficial effect of regional anesthesia on cancer outcomes in children. Despite this, regional anesthesia remains useful as part of a multimodal analgesia regimen and, as such, improves patient comfort and satisfaction (3).

## Opioids

Opioids are widely used in the treatment of both perioperative and cancer-related pain. Like regional anesthesia, it may be suggested that by reducing perioperative pain and stress, opioids should be immunostimulating with beneficial effects on cancer outcomes (38). Evidence from *in vitro* and animal studies appear inconsistent, with differing effects of opioids on the immune system demonstrated. Immunosuppressive opioids include fentanyl and morphine, while tramadol appears to augment natural killer cell proliferation. Buprenorphine, hydromorphone, and oxycodone appear to have a neutral effect on the immune system (39). Methadone improves the efficacy of cancer treatment agents in glioblastoma and leukemia by inducing cell death and apoptosis (39).

A Danish cohort study of 34,000 women with breast cancer found no correlation between opioid prescription and breast cancer recurrence during an 8-year follow-up period, irrespective of opioid type, duration of use, or cumulative dose (40).

With respect to evidence in children, a small retrospective study of 75 patients <19 years who underwent cytoreductive surgery with hyperthermic intraperitoneal chemotherapy found no statistically significant association between opioid consumption (total amount of opioid consumed during the entire admission) and recurrence-free survival (HR: 1.0, 95% CI: 0.99–1.03, *p* = 0.55) or overall survival (HR: 1.01; 95% CI: 0.99–1.03, *p* = 0.22) (41). The patients typically received a continuous infusion of fentanyl or sufentanil and an epidural infusion of bupivacaine 0.075% with hydromorphone at 2–5 mcg/mL.

## Blood transfusion

Blood transfusion-related immunomodulation is postulated to result in unfavorable cancer outcomes for patients, predominantly by enhancing the suppression of natural killer cells (42). Packed red cells are known to contain several biological substances that are implicated in immunosuppression and tumor promotion. These substances include biologically active cytokines, non-polar lipids, residual leukocytes, and a mixture of proinflammatory lysophosphatidylcholines which may stimulate the production of proinflammatory cytokines (43).

Original studies investigating the effect of perioperative blood transfusion on oncological outcomes suggested an association between allogeneic blood transfusion and increased cancer recurrence and mortality. However, these studies were predominantly retrospective in nature and widely disputed (44, 45).

A large Cochrane review from 2006, which included over 12,000 patients from 36 different studies, supported the hypothesis that perioperative blood transfusion is associated with an increased incidence of recurrence for patients with colorectal cancer. These findings were consistent across different tumor stages although less evident in more advanced stages, which was likely due to the inherent negative effect of advanced disease on the risk of recurrence. Perioperative blood transfusion and colorectal cancer recurrence yielded an odds ratio of 1.42 (95% CI: 1.2–1.67) against transfused patients (46).

A retrospective multicenter study of children undergoing surgery for neuroblastoma found that the intraoperative administration of erythrocyte concentrates was associated with a reduction in recurrence-free survival (HR: 7.59, 95% CI: 1.36–42.2, *p* = 0.004). However, overall survival was unaffected (HR: 5.37, 95% CI: 0.42–68.4, *p* = 0.124) (42).

The effects of the administration of autologous blood on oncological outcomes during tumor surgery remain unclear. A randomized controlled trial of 475 patients with colorectal cancer found no significant difference in prognosis between the allogenic transfusion group and the autologous transfusion group (47). This contrasts with a small retrospective study of 165 head and neck cancer patients, which found that recipients of heterologous blood had a 40% increased risk of cancer recurrence (48).

The known immunosuppressive effects of transfused blood, together with the recognized complications of blood transfusion, have led to the development and widespread implementation of patient blood management programs with the aim of avoiding unnecessary blood transfusion and improving patient safety and outcomes.

## Other agents

### NSAIDs

Cyclooxygenase is an essential enzyme in the production of prostaglandins. As prostaglandin production is augmented during periods of inflammation, it has been demonstrated that prostaglandins may have a role in cancer progression (49). It is thus suggested that NSAIDs may have both a preventative and therapeutic role in cancer development.

NSAIDs, both nonselective and selective COX-2 inhibitors, have a large body of evidence that supports their use, with a demonstrated reduced risk of occurrence and progression of colorectal cancer (50). This benefit needs to be considered in the context of significant associated side effects.

A Danish breast cancer study showed that the post-diagnostic use of aspirin or selective COX-2 inhibitors was not associated with a decreased rate of recurrence, but pre-diagnostic use was associated with a decreased rate of recurrence (51). This study was contradicted by another meta-analysis, which concluded that NSAID and aspirin use after, but not before, diagnosis was associated with improved breast cancer survival, including breast-cancer-specific mortality, all-cause mortality, relapse, and metastasis (52).

Regarding the perioperative administration of NSAIDs, a pilot study of 45 patients with prostate cancer randomized to celecoxib 400 mg BD or no treatment for 4 weeks before radical prostatectomy found that celecoxib decreased tumor cell proliferation, micro-vessel density, angiogenesis, and HIF-1 while enhancing apoptosis (53).

NSAIDs are widely used perioperatively, and while there is a trend toward positive oncological outcomes in adults, there is an absence of evidence in children. This may be because perioperative NSAID use is rare in pediatric oncology patients due to the presence of a low platelet count or concerns about nephrotoxicity.

### Ketamine

Ketamine is widely used perioperatively for its analgesic properties. Its activity at various receptor sites ensures that it is a useful agent for both the management of acute postoperative pain and the management of chronic pain and opioid tolerance. Ketamine is predominantly a NMDA receptor antagonist with activity at GABA, acetylcholine, opioid, and monoamine receptors as well as sodium and calcium channels (54). While the analgesic effects of ketamine are well established, its effects on the immune system and, hence, cancer treatment remain unclear.

Clinical studies on the oncological outcomes associated with ketamine are scarce, even in adults. A prospective, randomized study investigating the effect of subanesthetic doses of ketamine on natural killer cell activity and inflammation in patients undergoing surgery for colorectal cancer found no difference in postoperative natural killer cell activity, inflammatory response, or prognosis with the use of low-dose ketamine (55). Similarly, a retrospective study found no favorable oncological outcomes with the use of ketamine in breast cancer patients (56).

There is no evidence available on the oncological outcomes associated with ketamine usage in children.

### Alpha-2-agonists

Clonidine has a long-established perioperative role in children. More recently, dexmedetomidine use is also gaining popularity. These agents, through their downregulation of the sympathetic response, are used for their analgesic, anxiolytic, and sedative effects.

A limited number of *in vitro* studies suggest that dexmedetomidine and clonidine may be associated with the augmented growth of tumor cells and metastases (57, 58).

Similarly, clinical studies on either immunological or oncological outcomes associated with the use of alpha-2-agonists are very scarce. A retrospective study of 1,404 patients found that the intraoperative use of dexmedetomidine in patients with non-small cell lung cancer was not associated with a significant impact on recurrence-free survival (HR: 1.18, 95% CI: 0.91–1.53, *p* = 0.199), with no difference demonstrated in 5-year recurrence-free survival rates between the two groups, but with reduced overall survival (HR: 1.28, 95% CI: 1.03 – 1.59, *p* = 0.024) (59).

A retrospective chart review evaluating the survival impact of dexmedetomidine in children undergoing cytoreductive surgery with hyperthermic intraperitoneal chemotherapy found that the administration of dexmedetomidine was not associated with progression-free survival (HR: 1.2, 95% CI: 0.6–2.4, *p* = 0.606) or overall survival (HR: 0.81, 95% CI: 0.35–1.85, *p* = 0.611) (60).

### Steroids

Steroids are routinely used for their cytotoxic, antiemetic, and immunosuppressive properties (61). Dexamethasone is not routinely administered for oncological patients at our institution, as it regularly forms part of the oncology treatment regimen. Administering steroids before getting a tissue diagnosis is also generally avoided as it may potentially interfere with the formal diagnosis. Furthermore, steroid use may risk precipitating tumor lysis syndrome in oncological patients (62).

## Conclusion

There is mounting evidence supporting an association between perioperative interventions such as anesthetic techniques and oncological outcomes in adults. Evidence suggests a trend toward an effect rather than a definitive answer. TIVA and NSAIDs in the perioperative period may be beneficial, while perioperative blood transfusion appears to be associated with more harm.

The significant differences between adult and pediatric oncology populations, in terms of immune system maturation, underlying malignancy, treatment regimens, and frequency of anesthetic exposure, make it difficult to extrapolate findings from adults to children. However, evidence from adults is important to consider as children share more physiological similarities with adults than with experimental animal models.

In addition, the potential impacts of anesthesia on pediatric oncological outcomes may be influenced by different cancer types, stages, and even treatments. There is a distinct lack of data on this topic in the pediatric population, and it is therefore an important topic for future evaluation. A large, high-quality randomized, controlled trial is warranted to further our understanding of the effects of anesthesia in pediatric oncology patients.
